# Remarks on the Treatment of Intermittent Fever

**Published:** 1856-10

**Authors:** Edward Jenner Coxe

**Affiliations:** Visiting Physician, Charity Hospital, New Orleans


*Remarks on the Treatment of Intermittent Fever, and the Employment of the Sulphate of Cinchonia as a Substitute for Quinine.* By Edward Jenner Coxe, M. D., Visiting Physician, Charity Hospital, New Orleans.

The attention of physicians has been for years directed to the discovery of a substitute for the sulphate of quinine, thus far regarded as an unequalled remedy for the treatment of many diseases, besides intermittent and other fevers. Of the many articles submitted to the test of experience, the sulphate of cinchonia appears to have obtained the confidence of the profession to the greatest extent. This article is procured from several varieties of cinchonia, which yield quinine in small quantity.

Dr. W. Pepper, in the American Journal of Medical Sciences, for January, 1853, has recorded his successful trials of this preparation, and in other journals may be found reports equally favorable to the value of this curative agent. The sulphate of quinine being an expensive agent, often beyond the reach of those of small means, and apprehension being entertained, from its extensive and annually increasing use, that at some future day there may prove a difficulty in procuring a sufficient supply of the bark which most abundantly yields this valuable remedy, are sufficient reasons for noticing the subject.

Having used during the past year the sulphate of cinchonia, almost to the exclusion of quinine, in the wards of the Charity Hospital under my charge, and finding it in all respects equal as a curative agent to quinine,

I am of opinion that by extending the knowledge of its value, others may be induced more frequently to test its power, and report the result of their experience with it. During the time specified, I have treated upwards of one hundred cases of intermittent fever, in the wards 32 and 33, of every degree of violence and duration, with a degree of success perfectly satisfactory, and although a particular course of treatment was pursued, and other medicines were conjoined with the sulphate of cinchonia, still, as this sulphate was employed in every case, it may, I think, be fairly asserted, that the use of quinine was not required, and that as a substitute, the sulphate of cinchonia proved itself in all respects to that extensively used, and acknowledged remedy. The cases admitted were generally of some standing, varying from one or more months to several years, and as a consequence of the repeated attacks, or the result of an imperfectly cured disease, there was presented every degree of arrangement, and enlargement of the liver and spleen, with some cases of dropsy, and most generally that morbid physiognomy, indicating an impaired, or broken down state of health, the result of irregular habits, conjoined with this disease in a chronic form.

Upon questioning the patients, it generally appeared that the principal remedy upon which reliance had been placed to effect a cure, was frequent doses of quinine, with but seldom, any proper preparatory attention having been paid to the condition of the digestive organs, which in my opinion is indispensable to the effecting a certain and speedy cure. Admitting the fact, that quinine will frequently arrest a poroxysm of ague, and at times prevent a recurrence, it cannot be considered that the temporary suspension of a paroxysm, consisting of the cold, the hot, and the sweating stages, is identical with the overcoming of the disease, of which they are but the symptoms.

The question presents itself, whether at the present day, we do not more frequently meet with the secondary effects of ague and fever, as enlargement of the liver and spleen, dropsical affections, or a general deterioration of health, than was the case prior to the discovery, and introduction into popular use, of the valuable quinine, or whether, in consequence of its acknowledged power as a curative agent, it is not too exclusively trusted to without that preliminary treatment which all must admit to be of the utmost importance in effecting a lasting cure.

We meet with many cases of intermittent fever, of long continuance, in which, at times from the commencement of the attack, there will not have been a regular shake, but rather a sensation of coldness, or shivering, with lividity of skin and nails, followed by fever of greater or less severity and duration, terminating in profuse perspiration. This is known as the dumb ague, it is regarded as being difficult to perfectly cure, but has been equally successfully managed as the more constant forms by the same course of treatment. Before the discovery of quinine, when the cinchonia bark was considered the specific, it was an almost general rule to commence the treatment of intermittent fever by the exhibition of an emetic, and one of tartar emetic was commonly preferred, from its more decided action upon the general system. This, aided by copious draughts of warm water, or chamomile tea, would produce a free discharge of bile from the liver, and with it the entire contents of the stomach, accompanied generally by profuse perspiration. At the expiration of a few hours, generally at bed time, there was given a dose of calomel, whose certainty of action the following morning, was secured by one or more doses of oil, senna and salts, salts and magnesia, or other cathartic. The experience resulting from the use of so large a number of emetics consecutively in a given period of time in intermittent and other diseases, justifies, it appears to me, the assertion, either that unpleasant consequences do not so frequently result, as we should be induced to infer from the rarity of their use, and the apprehensions entertained of them by some physicians, or that the addition of the articles conjoined, had the power of preventing their appearance.

In pursuance of this course of treatment, at bed time, there was ordered from ten to fifteen grains of calomel, combined with eight or ten of Dovers' powder, or two or three of the modified blue pill, to be followed the next morning by a moderate dose of oil, or other cathartic, if required.

The sulphate of cinchonia was now brought into use most generally, as follows :

Rec. Cinchonia Sulph. ʒssad℈jj,
Syr. Rhei. ℥j,
Liq. Arsenici Fowlerii, ℥is,
Tr. Gentian. Comp. ℥iii,
Tr. Cinchon. Comp ʒiii*m*.

Dose, two tabespoonfulls every two hours during the day. A continuance of the same for a few days, with some rather longer, succeeded in overcoming the disease, which in all probability would not have been the case, had the preliminary remedies been omitted. In addition to this remedy, there was given during the day, at regular intervals, a rtrong infusion of the best tonics, as bark, snake root, calumbo, quassia and aromatics, and also in some cases a teaspoonful of a powder composed of iron, bark, ginger and cinnamon, two or three times a day, taken in the tonic drink.

After having fully verified the efficacy of the above combination, I used after the preliminary treatment, the sulphate of cinchonia, by itself, in doses of from ten to twenty grains two or three times a day, at times conjoining with it a few grains of Dovers' powder and rhubarb, as might appear indicated. The same results have followed its use in this manner, and it is worthy of remark, that in no one instance was there produced any of those unpleasant sensations, so constantly complained of, after even moderate doses of quinine, no uneasiness in the head, no ringing of the ears, no deafness. Occasionally, with the view of more thoroughly testing these points, it was given, when considered admissible, in the dose of two scruples, and in a few cases, of one drachm, with similar results.

The properties of the two medicines, being in other respects equal, this difference, if found when tried by others, to prove a constant rule, must certainly in many cases, give the sulphate of cinchonia a superiority over quinine. During the sickness and convalescence, the diet was principally of a farinaceous character, with, in some cases towards the end of the treatment, a small quantity of alcoholic or malt liquor. With hospital patients it is clearly impossible to assert that all would escape a return of the disease, some leaving as soon as apparently cured, but as many of the more severe cases remained in the house some time after the cure was effected, without the least appearance of a return, it is but fair to conclude that such was the case most frequently. In view of the above facts, I

think it may be asserted, that the sulphate of cinchonia has displayed curative powers, quite equal to those of quinine, and as there is a very material difference in the price of the articles, the promulgation of the above facts may induce others of the profession to test them in other places, and found to correspond in point of efficacy, in no trifling degree benefit the pecuniary condition of charitable institutions.

It may not be amiss to state, that in many cases, where it was requisite to strengthen the system, without regard to any particular disease, the sulphate of cinchonia has been given in conjunction with the ordinary tonic tinctures, with uniformly good effects.

A few words in conclusion, as to the necessity and immediate advantage of administering emetics in many diseases, may not be amiss. With few exceptions, there was discharged from the stomach one or two basins full of offensive bilious matter of every grade of color, and it is clear that had such remained in the system, until removed by purgatives, through the intestinal canal, not only would the cure have been materially prolonged, but the liability of exciting irritation or inflammation in the viscera, increased, far beyond what might occasionally result from any emetic that might be administered.—*N. O. Med. News & Hos. Gaz.*

